# Joint recommendations for a total services account as a factor in simplifying contracts

**DOI:** 10.3205/000276

**Published:** 2019-10-24

**Authors:** Insa Bruns, Carmen Schade-Brittinger, Frank Wissing, Thorsten Ruppert, Martin Trillsch

**Affiliations:** 1 Coordination Center for Clinical Studies (KKS-Netzwerk e.V.), Berlin, Germany; 2 Coordinating Centre for Clinical Trials Marburg (KKS Marburg), Philipps University Marburg, Germany; 3 German Association of Medical Faculties (MFT), Berlin, Germany; 4 German Association of Research-Based Pharmaceutical Companies (vfa), Berlin, Germany; 5 German Association of Academic Medical Centers (VUD), Berlin, Germany

## Abstract

The objective of clinical trials is to transfer findings gained from basic research to patients and to result in innovative treatment approaches. Along with basic research, results from clinical trials thus represent a core area of medical advances. As a location for clinical trials, Germany is currently well-positioned and internationally competitive. This is evident in its position as No. 2 in Europe and No. 3 worldwide – behind the US and UK – in clinical trials of pharmaceuticals [1]. Maintaining and further improving this favorable positioning as a location for clinical trials is in the mutual interest of all parties involved in the field of clinical research, patients, trial sites and sponsors of clinical trials.

For patients, clinical trials offer opportunities to gain early access to innovative therapy options. In addition to the scientific interest from medical faculties, clinical research is thereby an important aspect for university clinics in Germany as they fulfill their medical care mandate. Their involvement in clinical trials gives physicians the ability to gather experience with new treatment approaches at an early stage and to pass this know-how on to their patients. A location’s clinical research is thus an important competitive factor in terms of international comparison as well. Industry likewise benefits from the favorable research infrastructure in Germany, which provides rapid patient recruitment and outstanding quality of results obtained and can thus contribute to the early approval of new drugs. From the perspective of the authors, it is therefore essential that Germany continues to remain competitive as a location for conducting clinical trials, precisely because the number of clinical trials is decreasing overall. Companies themselves are in international competition internally and externally, which often creates a certain pressure on trial preparation and thus on the start of a clinical trial. To ensure that a clinical trial can begin early, it is essential that contracts related to the trial are concluded quickly and simply, including remuneration for participants and full, transparent and comprehensible coverage of content for the business relationship. The swift agreement of key contractual and budgetary aspects is therefore in the interest of everyone involved.

Against this backdrop, the German Association of Medical Faculties (MFT), the German Association of Academic Medical Centers (VUD), the Coordination Center for Clinical Studies (KKS-Netzwerk) and the German Association of Research-Based Pharmaceutical Companies (vfa) have held joint discussions regarding an important aspect of the contract negotiations – the cost consideration of clinical trials.

As a result of these talks, these organizations have developed and published joint “Recommendations for the preparation of a total services calculation for remuneration related to the conduct of a clinical trial in a trial center” [2], [3]. The parties concerned share the conviction that, against the backdrops described, it would be helpful if the potential contract partners had access to recommendations that offer examples of constantly recurring cost positions in order to more precisely determine remuneration related to the conduct of a clinical trial.

This article explains how the “Recommendations for the preparation of a total services calculation for remuneration related to the conduct of a clinical trial in a trial center” [2], [3] were developed and provides an overview of their content.

## Zusammenfassung

Klinische Prüfungen haben zum Ziel, die in der Grundlagenforschung gewonnenen Erkenntnisse auf den Patienten zu übertragen und innovative Behandlungsansätze zu schaffen. Neben der Grundlagenforschung bilden die Ergebnisse klinischer Prüfungen damit einen Kernbereich medizinischen Fortschritts. Derzeit ist Deutschland als Standort für die Durchführung klinischer Prüfungen gut aufgestellt und international wettbewerbsfähig. Dies zeigt sich an seiner Position als Nummer 2 in Europa und Nummer 2 weltweit – hinter den USA bzw. UK – bei klinischen Prüfungen von Arzneimitteln [1]. Diese gute Positionierung des Studienstandortes zu erhalten und weiter zu verbessern, liegt im gemeinsamen Interesse aller Beteiligten im Bereich der klinischen Forschung, der Patienten, der Studienzentren und der Sponsoren klinischer Prüfungen.

Für Patienten bieten klinische Prüfungen die Möglichkeiten, frühzeitig Zugang zu innovativen Therapiemöglichkeiten zu erhalten. Neben dem wissenschaftlichen Interesse der medizinischen Fakultäten ist die klinische Forschung damit für die Universitätskliniken in Deutschland ein wichtiger Aspekt bei der Wahrnehmung ihres Auftrags der Krankenversorgung. Die Einbindung in klinische Prüfungen gibt den Ärzten frühzeitig die Möglichkeit, Erfahrungen mit neuen Behandlungsansätzen zu sammeln und an die Patienten weitergeben zu können. Klinische Forschung an einem Standort ist damit ein wichtiger Wettbewerbsfaktor auch im internationalen Vergleich. Die Industrie profitiert auf der anderen Seite von der guten Forschungsinfrastruktur in Deutschland, da diese einen schnellen Patienteneinschluss und eine sehr hohe Qualität der gewonnenen Ergebnisse gewährleisten und damit zu einer frühzeitigen Zulassung eines neuen Arzneimittels beiträgt. Aus Sicht der Verfasser ist es daher wichtig, dass der Standort Deutschland bei der Durchführung klinischer Prüfungen auch zukünftig wettbewerbsfähig bleibt, gerade weil die Zahlen klinischer Prüfungen insgesamt zurückgehen. Die Unternehmen stehen ihrerseits im internationalen Wettbewerb, so dass die Vorbereitung der Studiendurchführung und damit der Start einer klinischen Prüfung häufig einem gewissen zeitlichen Druck unterliegen. Um eine klinische Prüfung frühzeitig beginnen zu können, ist es unerlässlich, Studienverträge inklusive der darin enthaltenden Vergütung zwischen den Beteiligten schnell und einfach, dabei aber inhaltlich umfassend, transparent und nachvollziehbar in der Leistungsbeziehung abzuschließen. Die zügige Einigung der wesentlichen vertraglichen sowie budgetären Aspekte liegt damit im Interesse aller Beteiligten.

Vor diesem Hintergrund haben der Medizinische Fakultätentag (MFT), der Verband der Universitätsklinika Deutschlands (VUD), die Koordinierungszentren für Klinische Studien (KKS-Netzwerk) und der Verband forschender Arzneimittelhersteller (vfa) gemeinsam einen wichtigen Aspekt im Hinblick auf die Vertragsverhandlungen diskutiert – die Kostenbetrachtung klinischer Prüfungen.

Als Ergebnis dieser Diskussionen haben diese Organisationen „Gemeinsame Empfehlungen zur Erstellung einer Gesamtleistungsrechnung der Vergütung bei der Durchführung einer klinischen Prüfung in einem Prüfzentrum“ erarbeitet und veröffentlicht [2], [3]. Die Beteiligten sind gemeinsam der Überzeugung, dass es vor dem beschriebenen Hintergrund hilfreich erscheint, wenn die potentiellen Vertragspartner in ihren jeweiligen Verhandlungen über Empfehlungen verfügen, die stetig wiederkehrende Kostenpositionen zur näheren Bestimmung der Vergütung im Rahmen der Durchführung einer klinischen Prüfung beispielhaft benennen.

Dieser Artikel erörtert die Hintergründe der „Gemeinsamen Empfehlungen zur Erstellung einer Gesamtleistungsrechnung der Vergütung bei der Durchführung einer klinischen Prüfung in einem Prüfzentrum“ [2], [3] und ermöglicht einen Überblick über deren Inhalt.

## Baseline

Conducting patient-oriented clinical trials is of tremendous importance. Clinical research is an essential requirement for the successful development and adoption of new pharmaceuticals and forms of therapy and constitutes an important foundation of evidence-based medical care. Clinical trials improve the quality of physician treatment and provide the necessary requirements and decision certainty for the efficient use of pharmaceuticals. In order to safeguard the quality of clinical trials, they must be conducted in compliance with strict national and international guidelines. These concern both patient care and safety and the qualification of physicians and participating clinics, one example being the regulation requiring implementation of good clinical practice (ICH-GCP) in the conduct of clinical trials on medicinal products for human use.

The investments from university medicine faculties in Germany, and in particular the supporting programs of the Federal Ministry of Education and Research (BMBF) that established the Coordination Center for Clinical Studies (KKS) and subsequent funding for clinical trial sites have boosted the competitiveness of Germany as a location. In addition, the approval of requests for clinical trials launched EU-wide in 2004 is carried out in Germany in a proper and timely, scientifically sound manner by the higher federal authorities, the Federal Institute for Drugs and Medical Devices (BfArM), and the Paul Ehrlich Institute (PEI). The current system of rating multi-center clinical trials by a leading ethics commission, in place since 2004, has proven itself successful for the most part, even if the details show some need for improvement (e.g. further balancing of requirements from individual ethics commissions, reducing bureaucratic requirements).

Germany, currently the third largest market for the pharmaceutical industry worldwide, offers favorable conditions overall for efficient patient-oriented research. With 2.3 medical specialists per 1,000 residents, Germany has the highest concentration of such medical specialists compared to other industrialized nations [1], [4] a factor that represents an important condition for conducting clinical trials. Germany’s relatively high population density with an associated higher-than-average number of patients in the vicinity of very well-equipped medical care facilities, such as university clinics, is another favorable basic condition for patient-oriented research. However, Germany is considered an attractive location for clinical trials first and foremost because of the high quality standards that exist in research and the fundamental scientific know-how of its practicing physicians.

Together, these factors contributed to Germany taking the No. 2 position in Europe and No. 3 position in the world behind the USA and in Europe from 2007 to 2016 as No. 1 in Europe, falling behind the UK just in 2017 – based on the number of clinical trials held [1]. In an international ranking, Germany remains in position No. 2 with 7,359 trial sites; it is behind the USA (49,472) and far ahead of its international and European competitors (France (4,628), Canada (4,186), Italy (3,246), and UK (2,866) [4]).

Maintaining and further improving critical aspects of Germany’s favorable positioning as a place to conduct studies is a shared concern of all interested parties and was the reason for discussions between the German Association of Medical Faculties (MFT), the German Association of Academic Medical Centers (VUD), the Coordination Center for Clinical Studies (KKS-Netzwerk), and the German Association of Research-Based Pharmaceutical Companies (vfa).

In connection with these talks, the associations identified two areas which, in their view, could contribute to substantial shortening of contract negotiations before clinical trials: preparation of templates for contractual clauses and a mutual understanding of the total services account. The parties worked together on both aspects.

The starting point in the discussion surrounding the total services account was the varying experience of the partners involved, which is briefly summarized from the perspective of the relevant partner and presented as follows.

## Current problems with the total services calculation for remuneration of sites

From the point of view of the trial sites, coverage of overhead costs or site-specific costs is not always guaranteed. If these costs are not accounted for in the course of clinical trials, the trial sites are not guaranteed remuneration that fully covers costs as is provided for in the state aid law. The consequence is underfunding of clinical trials that support the research infrastructure overall. In the medium term, insufficient financing of clinical trials that support research infrastructure could lead to the risk of diminished quality and thus a weakening of Germany as a place to conduct studies. To date, for reasons of administrative simplicity, the trial sites have billed the costs as a lump sum overhead charge.

From the point of view of industrial sponsors, a lump sum settlement of mutual costs as overhead is problematic because the basis for their calculation does not depict the actual business relationship with sufficient transparency. When individual locations negotiate separate contracts with separate budgets for trial-based services (e.g. radiology, pharmacy, etc.), scheduling delays are often the result. In addition, claims are increasingly made in the form of initiation costs (often referred to as set-up costs). The member companies consider these claims to be acceptable only if they correspond with the services actually rendered, not when they are charged as a ‘lost opportunity’ in the event of a terminated trial or lack of recruiting. Member companies of the vfa consider time delays in trial preparation and non-transparent cost calculations in particular to be competitive drawbacks for conducting clinical trials in Germany.

The aspects mentioned are increasingly topics that require discussion, which can lead to extended contract negotiations and thus a delay in the start of the trial. The high quality of research achievements in connection with clinical trials in Germany is very attractive in the international competitive field and must be utilized. The objective of the participants was therefore to ensure that contract negotiations do not unnecessarily delay trial preparation, which could lead to trials being relocated to other regions. All participants hope that the joint recommendations for the preparation of a total services calculation for remuneration related to the conduct of a clinical trial in a trial site will further strengthen Germany as a place to conduct studies overall.

## Joint recommendations

Against this backdrop, in mid-2016, representatives from the German Association of Medical Faculties (MFT), the German Association of Academic Medical Centers (VUD), the Coordination Center for Clinical Studies (KKS-Netzwerk), and the German Association of Research-Based Pharmaceutical Companies (vfa) came together to discuss cost trends of clinical trials. The parties considered it wise to prepare joint “Recommendations for the preparation of a total services calculation for remuneration related to the conduct of a clinical trial in a trial center” as a basis for future collaboration [2], [3]. The recommendations were worked out jointly in multiple meetings and intensively discussed with respective members of the involved associations in parallel.

The parties agreed that financing for all extra expenses arising from the conduct of a clinical trial must be ensured. This also includes the research infrastructure required for the trials. There was also agreement that, pursuant to the ban on double billing, no additional remuneration would be made for standard of care activities that were already reimbursed by health insurance funds – even if the patient is enclosed in a clinical trial. Another issue raised here is that of limiting trial-related additional expenses. Clarification is therefore needed to determine which of the measures performed when conducting a clinical trial according to protocol would be a required part of standard treatment and which are performed specifically in connection with the conduct of the clinical trial.

On this basis, the services to be remunerated by the sponsor include all services in direct connection with the clinical trial that are provided to or with the patient, such as a consultation (patient information), blood draws, physical and instrumental diagnostics, ECG, etc., and the preparation of necessary trial-specific documentation (reports, certificates). The patient information meeting, explanation and evaluation of questionnaires can thus be included. It might be necessary to divide remuneration into individual services per visit and to pay only for the service rendered. It should be determined in advance which services will be remunerated for patient screening if, after the screening, the patient is not accepted into the trial. A false incentive to take part in a “pointless” screening, caused by a too generous remuneration of screening services, should be avoided. Yet an insufficient remuneration of needed screening efforts actually undertaken should be avoided as well.

Costs for inpatient admission to hospital are to be assumed by the sponsor only if these are necessary for the proper and safe conduct of the trial according to the trial protocol. In addition to patient and treatment-specific expenditures, other costs also need to be considered; these are likely be covered by lump sum payments and include items such as electricity costs, telephone/fax/Internet, office supplies and the use of other materials and facilities. This might also include internal transportation costs for patients, such as to the diagnostic center if x-ray exams are needed.

Figures for some of these infrastructure costs (indirect costs/general expenses) can only be estimated, since documenting and verifying individual charges would require an unreasonably great effort. However, estimated amounts must still be realistic, verifiable and comprehensible and be adequately proportional to the length of time of the clinical trial, as well as comply with local market prices. The amount should also take into account costs estimated for rooms and equipment use. This includes items such as rooms for patient consultation and rooms provided for clinical monitors. These rooms are often reserved by clinics and used outside of clinical trials as well. It is not disputed that the use must be compensated; however, settling the proportional costs in a transparent and comprehensible manner with reasonable administrative effort on a case-by-case basis is not a trivial issue.

By agreeing on the joint “Recommendations for the preparation of a total services calculation for remuneration related to the conduct of a clinical trial in a trial center” [2], [3] we were able to formulate a basis that is mutually accepted by both parties (university trial sites and sponsors of commercial clinical trials).

If the respective trial protocol and the present joint recommendation are considered, they should help facilitate comprehensive remuneration of all trial-related costs in connection with a total services account based on the principle of payment for services rendered. The recommendations should be regarded as supplementary to the payments/activities listed in the trial protocol. On the one hand, the core of the recommendations is to provide guidance for precisely calculating costs derived directly from the trial protocol. In addition, indirect costs/general expenses are to be reflected in the respective employee rates. Along with the purely tariff-based remuneration, these should take into account calculated indirect costs/general expenses, as is customary for other services. Site-specific indirect costs/general expenses and possibly other operational costs must be considered in the respective total services account, provided that they are directly related to the conduct of the clinical trial. From the point of view of the partners it is also clear that no other percentage lump sum surcharge (overhead) should be applied.

In any event, care should be taken to ensure that the test site is remunerated in full for the service rendered. Participation in clinical trials cannot and must not be a loss-making business for university trial sites.

The following aspects and their effect on the total services account should be observed when implementing the joint recommendations.

- Various members of a trial group may be involved simultaneously in the activities listed.
- Activities *before* completion of a written contract are not covered by these recommendations. For these activities, additional remuneration can be agreed in a separate written contract (e.g. in the case of separate screening of the patient population in the context of expensive feasibility activities).
- The activities/tasks listed in the recommendations must be checked in each case for their relevance to the respective clinical trial. Remuneration based on the total services account is performed according to the principle of payment for services rendered.
- The total remuneration for a clinical trial project will be proposed on the basis of the total services account. This total remuneration also includes the trial-related services of all sub-contracted service providers, e.g. radiology departments or pharmacies. This proposal forms the basis for contract negotiation.
- Costs that are covered by other cost centers (e.g. statutory health insurance) – such as costs of standard treatment/diagnostic tests – cannot be recovered again/additionally with regard to a clinical trial.
- These joint recommendations cannot be binding due to antitrust legislation, but they are intended to be a jointly adopted guideline for the negotiation of clinical trial contracts by members of the above-mentioned organizations and other third parties.
- The recommendations include tasks that may be performed depending on the project in the context of conducting a clinical trial, and are not (or only insufficiently) described in relevant service specifications.

## Conclusion

Clinical trials are an essential requirement for the development and adoption of new pharmaceuticals and forms of therapy. They constitute the foundation for evidence-based medical care. Germany is an important and attractive location for conducting clinical trials.

The authors of these joint recommendations [2], [3] are united by their interest in boosting Germany’s international positioning as a location for clinical trials and in preserving the high standards established in Germany in the future as well. In this connection, it is essential to provide financing for clinical trials that is appropriate, comprehensive and aligned with the principle of adequate payment for services rendered. The joint recommendations developed by the German Association of Medical Faculties (MFT), the German Association of Academic Medical Centers (VUD), the Coordination Center for Clinical Studies (KKS-Netzwerk), and the German Association of Research-Based Pharmaceutical Companies (vfa) provide a sound basis for discussions in connection with contract negotiations related to the conduct of clinical trials at university clinics in Germany.

## Notes

### Acknowledgments

The joint “Recommendations for the preparation of a total services calculation for remuneration related to the conduct of a clinical trial in a trial center” were formulated in a joint work group in 2016/2017 and are available in English [2] and German [3]. Members of the work group included:

- Insa Bruns; KKS-Netzwerk e.V.†
- Susanne Busta; Bristol-Myers Squibb GmbH & Co. KGaA
- Dr. Annette Grüters-Kieslich; Charité Berlin
- Ralf Heyder; German Association of Academic Medical Centers (VUD)
- Dr. Torsten Hoppe-Tichy; University Hospital of Heidelberg
- Dr. Sebastian John; Janssen-Cilag GmbH
- Dr. Matthias Klüglich; Boehringer Ingelheim Pharma GmbH & Co. KG
- Claudia Küchler; MSD SHARP & DOHME GMBH
- Nina Reinwald; University Hospital of Cologne
- Dr. Thorsten Ruppert; German Association of Research-Based Pharmaceutical Companies e.V.
- Dr. Hagen Russ; Lilly Deutschland GmbH
- Carmen Schade-Brittinger; KKS Marburg
- Martin Trillsch; University Hospital of Heidelberg, VUD
- Dr. Frank Wissing; German Association of Medical Faculties e.V. (MFT)

### Obituary Insa Bruns

On January 21, 2018, Insa Bruns passed away after a brief and serious illness. The members of the work group that developed these recommendations are shocked and stunned at the sudden loss of such an esteemed colleague who was always highly dedicated and driven. We are all very grateful for her expertise and her tireless dedication. Her expert knowledge and perseverance in our joint endeavor to support high-quality medical research were much appreciated by all. Insa Bruns made a very valuable contribution to the issues of clinical research in Germany. She also became a personal friend to many of us over the years.

### Competing interests

The authors declare that they have no competing interests.
